# First Steps towards Evidence-Based Preventive Home Visits: Experiences Gathered in a Swedish Municipality

**DOI:** 10.1155/2012/352942

**Published:** 2011-07-21

**Authors:** Charlotte Löfqvist, Staffan Eriksson, Torbjörn Svensson, Susanne Iwarsson

**Affiliations:** Department of Health Sciences, Faculty of Medicine, Lund University, 221 00 Lund, Sweden

## Abstract

The purpose of preventive home visits is to promote overall health and wellbeing in old age. The aim of this paper was to describe the process of the development of evidence-based preventive home visits, targeting independent community-living older persons. The evidence base was generated from published studies and practical experiences. The results demonstrate that preventive home visits should be directed to persons 80 years old and older and involve various professional competences. The visits should be personalized, lead to concrete interventions, and be followed up. The health areas assessed should derive from a broad perspective and include social, psychological, and medical aspects. Core components in the protocol developed in this study captured physical, medical, psychosocial, and environmental aspects. Results of a pilot study showed that the protocol validly identified health risks among older people with different levels of ADL dependence.

## 1. Background

Old people's health and wellbeing are urgent questions for the society of today, and ageing in place is not only a common policy but also prioritised by the majority of older persons, in Sweden as in most European countries [1]. It is of great concern that this age group receives support during the process of ageing, and evidence-based health-related interventions are important to meet the needs of health and social care in this increasing population segment. In order to develop more efficient practices, the evidence base should be taken into account, integrating practical expertise and experience with the best available scientific evidence [2]. 

Preventive home visits targeting community-dwelling older persons represent one example of proactive societal action that has received growing attention. The purpose of PHV is to promote overall health and wellbeing in old age, to identify people at risk for health problems, to prevent further decline, to enhance the possibility for the individual to maintain activity and participation, to be in control of everyday life, and to experience life satisfaction [3–6]. Preventive home visits have attracted political attention, and, for example, in Denmark since 1998, such activities are mandatory by law. In Sweden, the government has allocated economic resources to all municipalities, encouraging the provision of PHV to all citizens aged 75+. Still, even if a knowledge base on research is available, few of the local initiatives are based on science and best practice. Based on an attempt to support a Swedish municipality in developing evidence-based PHV, the overriding purpose of this paper is to describe a methodological process generating recommendations for further research and practice implementation. 

In the scientific literature, PHV have been described as a dynamic process between the home visitor and the visited person, aiming to sustain and improve the older person's wellbeing and independence [7, 8]. According to Danish experiences, the older person should be seen in a social and a psychosocial context, involving family and friends. That is, the Danish approach to PHV is to give equal attention to needs for health services and social support, and to risks to loose control/independence in life [9]. However, the results and effects of PHV, analysed in several review articles, are mixed and difficult to compare [3, 8, 10, 11]. Besides the fact that older persons constitute a heterogeneous group, another reason for the divergent picture of results is that there is a lack of common definitions within the field. Different aims, methods, outcome measures, and designs of the preventive efforts have been used and described, and different disciplines value prevention differently [12]. 

For example, Van Haastregt and coworkers found no clear evidence of positive effects of PHV when the visits were not tailored to the older person's needs [8]. In contrast, Byles identified improved health in their review, but argued that it is hard to identify the underlying mechanism for successful outcomes [3]. Effects found by others were reductions in mortality, functional decline, and admissions to long-term institutional care and hospitals [13–16]. Further positive tendencies of health effects seen were related to activities of daily living (ADL), physical capacity, falls, and social activities [9, 17, 18], as well as aspects of participation and life satisfaction [5, 19]. These somewhat inconclusive results still indicate that the use of a multidimensional approach including medical, psychosocial, functional, and environmental areas seem to increase the possibilities for successful preventive effects. Other aspects of a successful intervention were that several home visits with each individual are necessary [4], and that training of the professionals involved is important [20]. Still, the overall effect of PHV is uncertain and valued in divergent ways. As yet, there is limited evidence regarding the content, design, and structure of successful approaches to PHV and the literature on how to successfully operationalize the existing knowledge base is scarce. Therefore, it is crucial to develop and evaluate efficient structures for PHV, in order to ensure efficient quality development based on scientific evidence and best practices. 

The aim of this study was to describe the first steps of the development of evidence-based PHV targeting independent-living community-dwelling older persons in a Swedish municipality. The specific aims were to

- identify the existing best evidence base for a PHV protocol,
- develop and present the content of a protocol,
- pilot the developed protocol, that is, describe the piloting procedures and present results in terms of face and convergent validity, feasibility, and sensitivity for the detection of health problems.

## 2. Methods

Starting out from Sacketts's definition of evidence-based practice [2]; that is, the integration of practical experiences and best available scientific evidence, this project was composed of different parts (Table 1). The development-evaluation-implementation process as outlined by Craig et al. [21] served as the methodological framework of our study. It comprises structured guidelines serving to help practitioners and researchers to systematically recognise and adopt appropriate methods when developing, evaluating, and implementing complex interventions to improve health. A key message is to give weights to the development and implementation phase of the intervention, as well as to the evaluation, not necessarily following a linear sequence. The current study was concentrated on the development of the content and design of a PHV protocol, followed by a first pilot test. Additional and necessary steps such as evaluation and implementation are not within the scope of the present paper.

### 2.1. Project Context and Organisation

The study was carried out in a large municipality in southern Sweden (126,000 inhabitants; 17% aged 66+, 5% 80+). The study was initiated by the Department of Social Care of older people in the municipality, and carried out in cooperation with researchers at Lund University (authors). In the municipality, there were prior experiences within the social care sector of providing PHV through a project financed by governmental development grants. The target population for the PHV activities was community-living persons in ordinary housing, 80 years of age or older, and independent of health care or social services from the municipality for their daily activities. At the prospect of the current study, municipality administrators and politicians decided to raise their ambitions and explore the possibilities to introduce an evidence-based approach to PHV. Subsequently, a formal agreement between the university and the social services administration was made, involving an administrator at the preventive unit within social care and a senior scientist (last author) at the university as the responsible parties. 

A project group was established, consisting of experienced social carers employed in the Department of Social Care of older people in the municipality and researchers representing physiotherapy, occupational therapy, and gerontology at the university. One of the researchers was appointed project leader (second author). Two advisory boards were established; one in the municipality and one in the university. An internationally acknowledged scientific expert in research on PHV served as an external consultant to the project. The intention of the PHV to be developed was that the PHV protocol should be possible to introduce in everyday work in Swedish social care municipality contexts. The preparation phase targeted financing and collaboration issues, prior to the formal agreement. 

### 2.2. Procedure

The process described in this paper consisted of three phases: (1) synthesis of the evidence base for PHV; (2) development of a protocol for PHV; (3) piloting of the PHV protocol, followed by revisions. 

#### 2.2.1. Synthesis of the Evidence Base for PHV

Initially, a review of randomized control trials (RCT), identified by means of two recent and comprehensive systematic meta-analyses [4, 16], was performed, applying a two-step procedure. After exclusion of four articles, due to lack of required outcome measures (data on health aspects or relocation, hospitalisation, and mortality) and shortcomings in the follow-up procedure of the PHV, 21 trials remained and were analysed by means of an exploratory approach. That is, the material was reviewed in depth, to classify each original trial as either having or not having a positive effect on health, and in this way we were able to identify factors associated with general importance of successful PHV programs when analysing all 21 trials together. Next, additional information from a set of state-of-the-art publications, that is, two cohort studies and one meta-analysis [4, 22, 23], was extracted and integrated in the evolving synthesis.

In order to capture viewpoints from the target group for PHV, two group discussions were performed, inspired by focus group interview methodology [24]. For one of the group discussions, older persons living in ordinary housing in the municipality, and with previous experience of PHV, were recruited in the following way: every tenth person that had been visited the year before was phoned and asked to participate until five persons, interested to share their experiences in a group discussion, had agreed (mean age 85 years). Participants for the other discussion group were recruited from different local organisations of senior citizens. Seven older persons volunteered, having an interest to share their views and ideas regarding PHV; none of those had any prior personal experience of PHV. The group discussions were performed at a local meeting point for seniors and lasted about two hours each. The discussions were initiated by giving information about previous PHV activities in the municipality. The project leader led the group discussions. Questions in the group sessions concerned views on the aims of a PHV, and health areas to be included. The group members shared personal experiences; pros and cons, timing of PHV, the preferred location for the interviews, how the home visits and interventions should be accomplished and designed, and the group to target. Notes were taken and confirmed with the participants by the group leader directly connected to each session. The participants' viewpoints were analysed by the group leader who summarised and condensed the content into areas relevant for PHV.

In addition, prior experiences of PHV within the social sector in the municipality were collated in a report, constituting descriptive data of persons receiving such visits. This description of the target group, their needs, and what information and services had been distributed in connection with the PHV served as an important input to the evidence base.

Utilising the different types of data and information thus collected in an integrated manner, the synthesised evidence base was subsequently established, involving the two projects groups in iterative discussions. In addition, the material was discussed in a seminar involving the external consultant and additional researchers at the university.

#### 2.2.2. Development of a Protocol for PHV

In the construction phase of the protocol of PHV, the evidence base that was defined (as described above) was taken into consideration and used. Researchers and municipality employees met on a regular basis in an iterative process, involving identification of different health assessments, followed by education and training in how to administer such instruments during PHV. The content of the protocol, its design, and level of structure were intensively discussed, as were possible scales and questions to be included. Moreover, the advisory boards as well as the external expert were involved in the development process.

The final, structured PHV protocol, described in the results section, consisted of questions and assessments for different health areas. Guiding instructions to the home visitor were included in the protocol. The protocol also included suggestions for possible interventions based on the information collected, but this part was not piloted and is thus not further accounted for in the current paper.

#### 2.2.3. Piloting of the PHV Protocol

In order to pilot the PHV protocol, a strategic sampling procedure was performed, aiming at identifying 20 persons, 80 years of age or older, representing different levels of independence/dependence in ADL. The sampling was accomplished by a comparison of the population register against the social services register, the latter showing persons in use of alarm and home services. The strategy was to involve both men and women, in different living situations and housing conditions. Most important, persons with different levels of ADL independence were to be included, assuming that person with less ADL capacity had more risk factors, that is, in order to be able to study whether the new PHV protocol was able to detect persons with decreased health and/or health risks, allowing for piloting also of the in-depth questions/assessments in the health areas included [25].

In total, 49 invitation letters were sent out to potential participants. Forty-two of them were possible to reach by telephone and asked to participate in the pilot study. Twenty-six declined participation (17 of those were single-living women), mostly due to lack of interest, or time, or due to having health problems. Those who accepted participation and gave informed consent during the phone call were asked questions from the ADL Staircase assessment [26]. Finally, the sample consisted of 16 persons (13 men and three women, median age 83 years, range 80–92), categorised into three groups: independent in ADL, *n* = 11; dependent in I-ADL only, *n* = 3; dependent in both I-ADL and P-ADL, *n* = 2. Seven persons were married, nine were single-living (six men, three women).

Formal ethical approval was granted by the regional ethical review board (Dnr: 2009/516). Written informed consent was given by each informant in the study, after receiving information on the possibility to withdraw from the study at any time without having to state a reason.

The home visits were accomplished by four different home visitors; two experienced social carers and two researchers (occupational therapist, physiotherapist) from the university. All home visits had the same structure, following the new PHV protocol. The time used for each home visit was registered, and the visitors also recorded viewpoints of the PHV protocol, based on their experiences of each home visit, including comments from the older persons themselves.

For the data analysis, two of the ADL groups were merged combining those being independent with those dependent in I-ADL, that is, all those persons that were independent in P-ADL and thereby represented the target group for PHV. The results of the assessments included in the PHV protocol were analysed by means of descriptive statistics. In order to assess convergent validity, the median number of identified health risks was calculated for each ADL level group and the relationship between ADL level and number of identified health risks was analyzed. Moreover, to assess the sensitivity of detecting health problems for each health area included in the protocol, the total number of risks within each health area was identified. Time use was accounted for in minutes. The qualitative data (visitor experiences) was organised in a scheme, categorising the viewpoints recorded in technical, practical effectiveness, and more comprehensive aspects. Likewise, viewpoints from the visited persons were analysed. The analyses of quantitative and qualitative data [27] formed the basis for a revised version of the PHV protocol, constituting the end product of the part of the project presented in the current paper.

## 3. Results

### 3.1. Synthesis of the Evidence Base for PHV

The review of RCT studies (*N* = 21) showed that there was a tendency favouring interventions targeting higher age groups. That is, in the seven trials targeting a population with a mean or median age of 80 years of age or older, four demonstrated positive effects on health, compared to four out of 14 of the trials targeting a younger population. In the eight trials where they reported making home visits according to need, five had a positive effect on health versus three out of thirteen in those who did not. It might be important to have two or more professions represented in the team of home visitors. Out of eight trials reporting two professions or more making home visits, half of them had positive effects versus one-third of those trials where only one profession was represented. Our analysis of the health areas related to health effects included in the screening (from a set of state-of-the-art publications), concluded that information on medical, social, psychological, functional capacity, and environmental aspects were areas vital to screen.

The results of the group discussions implied that PHV should be performed in the person's home, be open in nature, and the staff should have enough time in order to detect needs in terms of health risks. Those who had prior personal experience of PHV expressed the importance of continuity, that is, that the same person was making repeated visits in order to attain confidence. The visit should contain both information and guidance since the health care and social services organisations are hard to understand, and knowing were and whom to turn to was perceived as hard to grasp. Overall according to both groups of older persons participating in the discussions, knowledge is lacking about what help and assistance is possible to receive, and what possibilities for meetings and activities for older persons are available. Stigmatizing aspects in contacts with the municipality, in particular with the social sector, were pronounced as an interfering factor for accepting PHV. The participants also stated that attitudes among older people themselves have to be changed in order to succeed in having more people being prepared to accept PHV. 

### 3.2. The Protocol for PHV

Based on the synthesised evidence base, core components in the protocol consisted of health areas capturing physical, medical, psychosocial, and environmental aspects, described in detail in Table 2. The health areas chosen had similarities with those previously used for PHV in the municipality, but were extended, made more structured, and were based on established assessment instruments, and did also include new areas such as cognition, depression, and physical capacity. 

Each health area of the PHV protocol was introduced with an open question in order to start the discussion, followed by structured questions or assessments. That is, when a person at risk was identified by means of predefined cutoff levels, in-depth follow-up questions or assessments were administered. The aim of the in-depth questions/assessments was two-fold: to secure that the assessment had captured a problematic health area, and to guide forthcoming interventions (while as such not included in the current study). For each health area the PHV protocol comprised a manual to serve as guidance for the home visitor and to provide in-depth information, followed by a suggestion on how to interpret the assessment results. Since staff with different professional training could be involved in PHV in the practice context, the degree of structure of the protocol ended up higher than initially intended. As far as possible, well-established and valid assessments and questions were chosen for the protocol. These decisions were taken in order to strengthen validity and reliability aspects.

### 3.3. Piloting of the PHV Protocol

The time use for administering the new PHV protocol varied between 45–130 minutes (median 90 minutes), with no differences between the three ADL level groups. For those PHVs where the visited person qualified for and responded to in-depth questions/assessments (one or more), the median time use was 120 min (range 90–130). The visited persons regarded the time use as reasonable, as did the home visitors. 

In total, 48 health risks, within all health areas, were detected by the protocol (M = 3, SD ± 2,3), distributed on ADL, other activities, social contacts, pain, depression, falls, impaired cognition, physical capacity, sight, or hearing (Table 3). The number of identified risks within each health area in the total sample varied from 1 to 10. Those participants' independent in P-ADL had the highest proportion of their identified health risks referring to pain, fall, hearing, and physical capacity. The lowest proportion referred to, in all ADL-level groups, was social contacts, nutrition, depression, and cognition. 

In the total sample, the number of health risks per person, according to ADL level group, varied between 0 and 8, indicating that the protocol had sensitivity for detecting health problems. The median number of health risks identified per person was twice as high in the dependent I-ADL group compared to the independent, indicating the convergent validity of the protocol. The median numbers of identified risks in the four ADL level groups are presented in Table 4. 

#### 3.3.1. Revision of the PHV Protocol

The overall point of view from the visited persons was that the questions were good and easy to understand and answer, even though three persons stated that the questions had several limitations. For example, some of the questions were experienced as hard to answer, they lacked information concerning health services and leisure possibilities for older people, and they thought that discussions regarding married life or more extended questions on memory issues were missing. These viewpoints were strengthened by the fact that examples of areas not included in the PHV protocol (health services, supports to relatives providing care, and meeting places for older people) spontaneously surfaced and were discussed during the home visits.

After the piloting the home visitors made a concluding evaluation of the protocol. A number of additional issues, mostly related to the structure of the protocol, were discussed by the home visitors and suggested for revision. Their points of view revealed that in most part the protocol was feasible to handle. However, aspects such as the protocol being too comprehensive and hard to navigate were also expressed. Concerns were raised regarding loneliness, weight, and medication as such questions had been felt too direct to ask, and the assessment of parts of the private home environment for barriers had resulted in similar reactions. On the other hand, questions on drinking habits and medication were suggested to be asked to all participants, instead of just being part of in-depth sections. Questions on P-ADL were not considered optimal; it did not seem adequate to ask independent persons about their need of help. 

In accordance with the specific study aim to present the content of a protocol for PHV, the protocol was revised based on the piloting. Most adjustments and changes were made in the areas of ADL and falls. The revised version contained the same health areas as the version piloted (Table 2), however optimized. Moreover, a suggestion for the future was to place the visitor instructions and in-depth questions/assessments in a separate manual, in order to facilitate navigation in the PHV protocol.

## 4. Discussion

The process described in this paper was accomplished in close collaboration between Swedish municipality practice and researchers, producing results with the potential to foster the development of evidence-based PHV in similar settings [2]. Even if the current study was delimited and represents only the very first steps of a long-term comprehensive project, it constitutes a first step towards an RCT. Currently, even if there is a sound base of scientific literature on PHVs available, PHV projects are being carried out in Sweden and in other Western countries mostly based on experience-based knowledge and practice. Since the uptake of research results in practice contexts in health care and social services is known to be insufficient, even if the current study is minor in scope it is an example of interaction between practitioners and researchers that might prove efficient in the forthcoming development of PHV. 

Pilot testing of the PHV protocol indicated that the convergent validity is acceptable. That is, since the number of identified health risks increased with poorer health, indicated by the ADL Staircase, health risks can be identified with the PHV protocol, which is in accordance with the intention of PHV to older people living in the community [8]. The median number of health risks identified among participants independent in P-ADL is in line with previous studies [42, 44, 45]. Since the health condition and functioning of older people are not static conditions [46], preventing ill-health is an ongoing process. In this kind of long-term work, it is important to identify health areas that may be added over time [47]. The results from a previous study of PHV show that for each year, new major health problems were identified in approximately one-third of the older persons visited [48]. 

It is widely accepted that ADL is an important health aspect and has strong support as a valid indicator of health among older people, even thought it does not cover all aspects of health [49, 50]. Some disadvantages in the ADL-Staircase instrument [26] can, however, be discussed, even if the results indicate that the instrument can be used to identify health risks and differentiate health status among groups of older people. One disadvantage is that the scale is not very sensitive, since it is based on a crude assessment of dependence and independence. Previous research has shown that the discrimination between different levels of ADL ability can be improved by adding a self-rated assessment of the difficulty associated with the performance [28]. Since the target group of PHV is persons independent of municipality services, with a specific focus to prevent health problems, there is reason to consider revision of the applications of the ADL-Staircase in the PHV protocol to increase the sensitivity for detecting changes over time. While not yet tested, it is reasonable to assume that such an improved PHV protocol can be used to also identify additional health problems in individuals over time. Longitudinal studies are, however, required to determine this type of sensitivity. In a study such as this where the participants only were studied at one occasion, the fact that more risk factors were identified in a group of older people with worse health (dependent in I-ADL) than in a group with better health (independent in ADL) is, however, an indication that the protocol is sensitive to also identify changes over time, and even better after further optimization.

The diversity of methods used in the current study, as implied by the guidelines of developing and evaluating complex interventions [21], was valuable for strengthening the content of the PHV protocol. The synthesis of the evidence base so far accomplished has implications for how to design and structure forthcoming PHV studies. Our suggestion is that an intervention group should receive PHV, alongside a control group receiving “usual treatment” involving general written community information but no followups or tailored interventions. The PHV practice should involve a multiprofessional team of visitors and contain at least two home visits per person and year on a regular basis, screening each individual for health risks. It is important to be aware of the complexity of the health and social services and diversity of the population segment for which PHVs are targeted. The preventive work in the intervention group should focus on individually tailored interventions with close followups targeting the oldest population, that is, 80 years of age and above. Outcome variables for PHV recommended in recent literature are self-perceived health, difficulty and dependence in ADL, participation, hospitalization, empowerment, falls, and control beliefs [4, 16, 22, 23]. Presumably, there are gains to be made from also including nonmedical aspects to a greater extent in PHV interventions while in the search accomplished prior to our project, no such literature was identified. 

Despite considerable efforts, we did not succeed in recruiting a larger sample with sufficient diversity, validly reflecting the target population. The low number of participants in the pilot study is a serious limitation, especially since those being partly dependent in ADL were very few (*n* = 3). In addition, only three of the 16 participants were women, even though there are far more women than men in the population aged 80 or older. Consequently, the results from the pilot study should be interpreted with caution; they mainly serve to test the protocol. The protocol may serve as an inspiration for further work, however, additional development and testing are needed to achieve sufficient validity, taking into account the heterogeneity of the target population as well as the need for focusing on effects and outcomes of PHV. 

As to the feasibility of the protocol, it should be noted that none of the participants felt that the home visits took too long. In a previous Swedish study, demonstrating positive results of PHV, the duration of the home visits reported was 60–180 minutes [13], compared to 45–130 minutes in the present study, implying that our method is time efficient. Furthermore, none of the participants felt that the home visits were too intrusive even though they contained potentially sensitive issues. For example, it is worth noting that participants asked for more questions related to memory and married life in old age. A few of them hesitated to perform physical testing and to allow screening of accessibility in their home. It is, however, reasonable to expect some internal drop-out when using such a comprehensive protocol. Such drop-out does not necessarily imply that the area in question should be removed from the protocol, since there will always be questions or areas not suitable to certain individuals or occasions. Participants that sought information about health care, municipal services and voluntary work were high. This kind of information was also given in prior PHV activities in the municipality and should therefore be included.

In this paper, the home visits were performed by different health care and social services professionals, all accustomed to visit and talk to old persons and to identify problems in a nonstructured way. This might have influenced the results of the pilot study in the sense that the shortcomings in the structured protocol to some extent were compensated for, that is, by the interviewers' communications skills and sensitivity to old people's health and social needs. In the discussions that took place after the data collection, the diversity in professional background was regarded as an advantage. This is in accordance with the multidimensional approach for preventive home visits suggested by others [4, 8, 16]. It should be noted, however, that for a long time in Sweden, medical competence (physicians) is not part of the municipality organization and responsibility, but rests within the county council. Consequently, involvement of general practitioners or geriatrics would imply even greater complexity and challenges beyond what was possible in this small project.

Among those participants independent in P-ADL, a large proportion with health risks in the areas of physical capacity, falls, hearing, and pain were identified. Despite the caution needed in the interpretation due to the small sample size, we note that that the proportion identified is reasonable in comparison to previous studies [44, 51]. There is evidence that reduced physical capacity increases the risk of ill-health, but just asking older people themselves to estimate their physical capacity is not reliable [22]. Therefore, reduced exercise capacity was assessed by the SPPB [52] in this study.

Barely half of the participants experienced an increased risk of falling. The link between falls, and in particular fall injuries, and functional decline is well established [22]. The four main risk factors for fall involved in the in-depth fall assessment of the PHV protocol seem relevant since we also identified one or more of these risk factors among those assessed as having a risk of falling. Also, decreased ability to perform meaningful activities in life is known to negatively influence health [53]. Questions assessing considerable changes in the patterns of activities were therefore considered important to involve in the protocol. 

It is a challenging but very important task to bring research results into practical applications/activities [54]. Studies of this nature are both challenging and informative, not only for researchers but also for the staff whose daily routines are challenged when scientific results become known. Public administrations, such as municipalities, consist of several different organizational levels with different perspectives (political, administrative, professional, and client centered) [55], and it is obviously important to a study such as this that its goals are anchored at all levels, in order to make the intervention work in everyday practice. It also puts great demands on the researchers' ability to flexibly adjust the study to the prevailing conditions and at the same time managing the study efficiently. Even if the government has given municipalities in Sweden financial support to start PHV to older people, there are no requirements to base the activity on the best available evidence. This study demonstrates a way to initiate such work, where scientists and employers in a municipality context work together to prepare evidence base, and then develop methods to test research-based methodology in practice. It is a long and complex process to develop scientifically based instruments and procedures, test and evaluate complex interventions and then introduce them as part of everyday work [21] in a municipality. The study presented in this report represents one example of the very first phase in such a process.

## 5. Conclusions

Based on the results and experiences from the study and the evidence base identified, we suggest that PHV, a protocol for older people, should include health areas derived from a broad perspective and include social, psychological, and medical aspects. The PHV should be based on an interview format with open as well as structured questions that make it possible to conduct the visit and interview in a personalized way. We suggest that the protocol have one part with questions and assessments that are performed with all informants, while there are specific follow-up questions, assessments, and scales integrated into the protocol that are applied for every case where a health risk is identified. It is important to also convey information about community services, especially health care and social sciences. With the reservation that we tested the PHV protocol in a pilot study with only few participants in a nonrepresentative sample, our conclusion is that the PHV protocol developed and piloted can be used for identification of health risks among older people with different levels of ADL dependence. Most important, the PHV protocol seems to be sensitive enough to identify health risks also among older people in good health, that is, those who represent the target group of PHV in Swedish municipalities. In order to establish the validity of the protocol, further studies are needed.

## Figures and Tables

**Table 1:** Project description.

Project part	Method approach	Participants
Identifying the evidence base		
(i) Literature review	Exploratory approach	First and second author
(ii) Group discussions	With inspiration from focus group interview methodology	12 older people, first author
(iii) Previous experiences in study district	Descriptive	First and second author, municipality employees
(iv) Seminar	Discussion	External consult, research team

Construction of the PHV protocol		
(i) Group discussions on regular basis	Iterative process	Research team, municipality employees
		Advisory boards, external expert
(ii) Education and training		Second author, municipality employees

Pilot study	Empirical	16 older people, first and second author, municipality employees

**Table 2:** Contents in PHV protocol administrated in the pilot study.

Health area	Assessment	Rationale/source	Structured questions^a^ (q), *n*		Criterion for using in-depth questionnaire
			Basic q	In-depth q	
Descriptive questions			5		
ADL	ADL Staircase	Items selected from the ADL-staircase [26]. Questions on difficulty were added [28].	5	1	Having difficulties in one or more I-ADL.
Comfort in home	Usability in My Home (UIMH)	[29, 30].	10	0	
Activities/interests	Study specific	The relation between meaningful activities and wellbeing is well established [31]. Open ended q on possible changes in pattern of activities and interest were used.	3	0	
Exercise	Study specific	Absence of physical exercise has showed to increase the risk for functional decline. Structured q on level of physical activity [32–36].	4	0	
Social contacts	Study specific	Used as a predictor of functional decline [22].	4	5	Not having anyone to contact if necessary or expressing feelings of loneliness.
Pain	SF-36	Based on clinical experiences [22], SF-36 were used [37], in combination with one q on pain in the feet.	2	4	Expressing moderate or worse pain.
Depression	GDS4, GDS20	Used as a predictor of functional decline [22].	4	16	Suspected depression (short version; GDS4).
Falls	Study specific	Q for screening of increased fall-risk and in depth assessment of potential causes of falls were based on risk-factors for falls identified by Ganz et al. [38].	4	13	Having had a fall during last year in an everyday situation or having trouble with walking, balance, or moving.
Pain/physical tests	SPPB-S	*Part of SPPB-S, used as a predictor of functional decline and relocation to nursing home [22, 23]. SPPB-S [39] was used as part of the in-depth assessment of potential causes of falls.	*Practical test	Practical test	
Environmental barriers	Housing Enabler Screening tool	Used as part of the potential causes of falls [38]. Housing Enabler, entrance, and indoors sections [40].	36	0	
Cognition	MMT	The connection between cognitive and functional decline is strong [22]. Short and long version of MMT (Minimental test) were used [41].	9	11	Suspected dementia (short version of MMT).
Medication	75+ health assessments	Selected from an Australian guide for health assessments [42].	5	Open-ended questions	Use of >3 medications in combination with difficulties remembering, lack of a physician contact, or using medication for anxiety, distress, or sleeping disturbance.
Food, diet	MNA	Mininutritional assessment (MNA).	6	9	Risk for malnutrition (MNA).
Health (perceived)	SF-36	Perceived health is a well-established predictor for mortality [22, 43]. One item form the SF-36 was used [37].	2	0	
Vision and hearing	Study specific	Used as a predictor of functional decline [22]. Identified as an important health factor in group discussions	2	0	
Evaluation q to the informant	Study specific		5	na	
Evaluation q to the interviewer	Study specific		10	na	

^a^ Each section starts with an open question introducing the topic, that is,* How do you manage everyday activities in your home?* or *Do you feel comfortable in your home?*

na = not applicable.

**Table 3:** Number of identified health risks within each health area in the PHV protocol in the pilot study, in total and according to ADL-level group, *N* = 16.

Health area	Independent in ADL, *n* = 11	Dependent in I-ADL, *n* = 3	Independent in P-ADL, *n* = 14^a^	Dependent in P-and I-ADL, *n* = 2	Total sample, *N* = 16
ADL	1	1	2	2	4
Activities/interests	1	1	2	2	4
Social contacts	1	0	1	0	1
Pain	3	3	6	2	8
Depression	0	0	0	1	1
Falls	3	3	6	2	8
Cognition	0	0	0	1	1
Medication	1	2	3	1	4
Food/diet	0	1	1	0	1
Physical capacity*	4/9	0	4	0/1	4/13
Vision	1	1	2	0	2
Hearing	6	2	8	2	10

Summa	21	14	35	13	48

P-ADL = personal activities of daily living.

I-ADL = instrumental activities of daily living.

*The last figure states the total number of persons within the ADL-level group that performed physical tests, since not all persons did.

^a^The groups independent in ADL and dependent in I-ADL merged comprising all persons independent in P-ADL.

**Table 4:** Median number of health risks identified in the pilot study, according to ADL level group, *N* = 16.

ADL level group	Median	Min–max
Independent in P-ADL, *n* = 14	2.5	0–7
(i) Independent in ADL, *n* = 11	2.0	0–4
(ii) Dependent in I-ADL, *n* = 3	4.0	3–7

Dependent in P- and I-ADL, *n* = 2	6.5	5–8

P-ADL = personal activities of daily living; feeding, transferring, toileting, dressing, and bathing.

I-ADL = instrumental activities of daily living; cocking, transporting, shopping, and cleaning.

## References

[1] Fänge A, Ivanoff SD (2009). The home is the hub of health in very old age: findings from the ENABLE-AGE Project. *Archives of Gerontology and Geriatrics*.

[2] Sackett DL (1986). Rules of evidence and clinical recommendations on the use of antithrombotic agents. *Chest*.

[3] Byles JE (2000). A thorough going over: evidence for health assessments for older persons. *Australian and New Zealand Journal of Public Health*.

[4] Stuck AE, Egger M, Hammer A, Minder CE, Beck JC (2002). Home visits to prevent nursing home admission and functional decline in elderly people: systematic review and meta-regression analysis. *Journal of the American Medical Association*.

[5] Clark F, Azen SP, Carlson M (2001). Embedding health-promoting changes into the daily lives of independent-living older adults: long-term follow-up of occupational therapy intervention. *Journals of Gerontology B*.

[6] Beswick AD, Rees K, Dieppe P (2008). Complex interventions to improve physical function and maintain independent living in elderly people: a systematic review and meta-analysis. *The Lancet*.

[7] Avlund K, Vass M, Hendriksen C (2007). Education of preventive home visitors: the effects on change in tiredness in daily activities. *European Journal of Ageing*.

[8] Van Haastregt JCM, Diederiks JPM, Van Rossum E, De Witte LP, Crebolder HFJM (2000). Effects of preventive home visits to elderly people living in the community: systematic review. *British Medical Journal*.

[9] Vass M, Avlund K, Lauridsen J, Hendriksen C (2005). Feasible model for prevention of functional decline in older people: municipality-randomized, controlled trial. *Journal of the American Geriatrics Society*.

[10] Bouman A, Van Rossum E, Ambergen T, Kempen G, Knipschild P (2008). Effects of a home visiting program for older people with poor health status: a randomized, clinical trial in the Netherlands. *Journal of the American Geriatrics Society*.

[11] Markle-Reid M, Browne G, Weir R, Gafni A, Roberts J, Henderson SR (2006). The effectiveness and efficiency of home-based nursing health promotion for older people: a review of the literature. *Medical Care Research and Review*.

[12] Clark J (2001). Preventive home visits to elderly people. Their effectiveness cannot be judged by randomised controlled trials. *BMJ Journals*.

[13] Sahlen KG, Dahlgren L, Hellner BM, Stenlund H, Lindholm L (2006). Preventive home visits postpone mortality—a controlled trial with time-limited results. *BMC Public Health*.

[14] Elkan R, Kendrick D, Dewey M (2001). Effectiveness of home based support for older people: systematic review and meta-analysis. *British Medical Journal*.

[15] Gitlin LN, Hauck WW, Dennis MP, Winter L, Hodgson N, Schinfeld S (2009). Long-term effect on mortality of a home intervention that reduces functional difficulties in older adults: results from a randomized trial. *Journal of the American Geriatrics Society*.

[16] Huss A, Stuck AE, Rubenstein LZ, Egger M, Clough-Gorr KM (2008). Multidimensional preventive home visit programs for community-dwelling older adults: a systematic review and meta-analysis of randomized controlled trials. *Journals of Gerontology A*.

[17] Steultjens EMJ, Dekker J, Bouter LM, Jellema S, Bakker EB, van den Ende CHM (2004). Occupational therapy for community dwelling elderly people: a systematic review. *Age and Ageing*.

[18] Theander E, Edberg AK (2005). Preventive home visits to older people in Southern Sweden. *Scandinavian Journal of Public Health*.

[19] Jackson J, Carlson M, Mandel D, Zemke R, Clark F (1998). Occupation in lifestyle redesign: the well elderly study occupational therapy program. *American Journal of Occupational Therapy*.

[20] Vass M, Hendriksen C, Thomsen JL, Parner ET, Avlund K (2008). Preventive home visits to home-dwelling older people and hospital admissions: a municipality-randomised intervention trial. *European Journal of Ageing*.

[21] Craig P, Dieppe P, Macintyre S, Michie S, Nazareth I, Petticrew M Developing and evaluating complex interventions: new guidance. http://www.mrc.ac.uk/Utilities/Documentrecord/index.htm?d=MRC004871.

[22] Stuck AE, Walthert JM, Nikolaus T, Büla CJ, Hohmann C, Beck JC (1999). Risk factors for functional status decline in community-living elderly people: a systematic literature review. *Social Science and Medicine*.

[23] Gaugler JE, Duval S, Anderson KA, Kane RL (2007). Predicting nursing home admission in the U.S: a meta-analysis. *BMC Geriatrics*.

[24] Halkier B (2002). *Fokusgrupper*.

[25] Law M, Baum C, Dunn W (2005). *Measurement in Occupational Performance*.

[26] Sonn U, Hulter Åsberg K (1991). Assessment of activities of daily living in the elderly. A study of a population of 76-year-olds in Gothenburg, Sweden. *Scandinavian Journal of Rehabilitation Medicine*.

[27] Creswell JW (2003). *Research Design: Qualitative, Quantitative, and Mixed Methods Approaches*.

[28] Iwarsson S, Horstman V, Sonn U (2009). Assessment of dependence in daily activities combined with a self-rating of difficulty. *Journal of Rehabilitation Medicine*.

[29] Fänge A, Iwarsson S (1999). Physical housing environment: development of a self-assessment instrument. *Canadian Journal of Occupational Therapy*.

[30] Fänge A, Iwarsson S (2003). Accessibility and usability in housing: construct validity and implications for research and practice. *Disability and Rehabilitation*.

[31] Townsend EA, Polatajko HJ (2007). *Enabling Occupation II: Advancing an Occupational Therapy Vision for Health, Well-Being, & Justice through Occupation*.

[32] Colcombe S, Kramer AF (2003). Fitness effects on the cognitive function of older adults: a meta-analytic study. *Psychological Science*.

[33] Elward K, Larson EB (1992). Benefits of exercise for older adults: a review of existing evidence and current recommendations for the general population. *Clinics in Geriatric Medicine*.

[34] Daniels R, Van Rossum E, De Witte L, Kempen GIJM, Van Den Heuvel W (2008). Interventions to prevent disability in frail community-dwelling elderly: a systematic review. *BMC Health Services Research*.

[35] Tessier D, Menard J, Fulop T (2000). Effects of aerobic physical exercise in the elderly with type 2 diabetes mellitus. *Archives of Gerontology and Geriatrics*.

[36] Hillman CH, Erickson KI, Kramer AF (2008). Be smart, exercise your heart: exercise effects on brain and cognition. *Nature Reviews Neuroscience*.

[37] Sullivan M, Karlsson J, Bengtsson C, Furunes B, Lapidus L, Lissner L (1993). ’The goteborg quality of life instrument’—a psychometric evaluation of assessments of symptoms and well-being among women in a general population. *Scandinavian Journal of Primary Health Care*.

[38] Ganz DA, Bao Y, Shekelle PG, Rubenstein LZ (2007). Will my patient fall?. *Journal of the American Medical Association*.

[39] Guralnik JM, Ferrucci L, Simonsick EM, Salive ME, Wallace RB (1995). Lower-extremity function in persons over the age of 70 years as a predictor of subsequent disability. *The New England Journal of Medicine*.

[40] Carlsson G, Schilling O, Slaug B (2009). Toward a screening tool for housing accessibility problems: a reduced version of the housing enabler. *Journal of Applied Gerontology*.

[41] Folstein MF, Folstein SE, McHugh PR (1975). “Mini mental state”. A practical method for grading the cognitive state of patients for the clinician. *Journal of Psychiatric Research*.

[42] Newbury JW, Marley JE, Beilby JJ (2001). A randomised controlled trial of the outcome of health assessment of people aged 75 years and over. *Medical Journal of Australia*.

[43] Idler EL, Benyamini Y (1997). Self-Rated Health and Mortality: a review of twenty-seven community studies. *Journal of Health and Social Behavior*.

[44] Hébert R, Robichaud L, Roy PM, Bravo G, Voyer L (2001). Efficacy of a nurse-led multidimensional preventive programme for older people at risk of functional decline. A randomized controlled trial. *Age and Ageing*.

[45] Vetter NJ, Lewis PA, Ford D (1992). Can health visitors prevent fractures in elderly people?. *British Medical Journal*.

[46] Hardy SE, Allore HG, Guo Z, Dubin JA, Gill TM (2006). The effect of prior disability history on subsequent functional transitions. *Journals of Gerontology A*.

[47] Statement NIoHCDC (1988). Geriatric assessment methods for clinical decision-making. *Journal of the American Geriatrics Society*.

[48] Alessi CA, Stuck AE, Aronow HU (1997). The process of care in preventive in-home comprehensive geriatric assessment. *Journal of the American Geriatrics Society*.

[49] Mulrow CD, Gerety MB, Cornell JE, Lawrence VA, Kanten DN (1994). The relationship between disease and function and perceived health in very frail elders. *Journal of the American Geriatrics Society*.

[50] Millan-Calenti JC, Tubio J, Pita-Fernandez S (2009). Prevalence of functional disability in activities of daily living (ADL), instrumental activities of daily living (IADL) and associated factors, as predictors of morbidity and mortality. *Archives of Gerontology and Geriatrics*.

[51] Fabacher D, Josephson K, Pietruszka F, Linderborn K, Morley JE, Rubenstein LZ (1994). An in-home preventive assessment program for independent older adults: a randomized controlled trial. *Journal of the American Geriatrics Society*.

[52] Guralnik JM, Simonsick EM, Ferrucci L (1994). A short physical performance battery assessing lower extremity function: association with self-reported disability and prediction of mortality and nursing home admission. *Journals of Gerontology*.

[53] Wilcock AA (2005). Occupational science: bridging occupation and health. *Canadian Journal of Occupational Therapy*.

[54] Fixen D, Naoomen SF, Blasé KA, Friedman RM, Wallace F (2005). *Implementation Research: A Synthesis of the Literature*.

[55] Iwarsson S, Jernryd E, Rutstrom C, Boqvist A (2000). Methodologic support in habilitation and rehabilitation: communicative action between practice and science. *Journal of Allied Health*.

